# Supplementary material for: The accelerating loss and shifting dynamics of US tidal wetlands
Source: Nat Commun. 2026 May 19;17:4332. doi: 10.1038/s41467-026-71464-2 (PMC13187131; doi:10.1038/s41467-026-71464-2)
Supplement: Supplementary file 1 — Supplementary Information [file 41467_2026_71464_MOESM1_ESM.pdf]

Supplementary Materials

Title: The accelerating loss and shifting dynamics of US tidal wetlands

Authors: Xiucheng Yang[1,*], Shi Qiu[1], Kevin D. Kroeger[2], Zhiliang Zhu[3], Scott Covington[4], Nicholas J. Murray[5], Zhe Zhu[1,*]

Affiliations:

[1] Department of Natural Resources and the Environment, University of Connecticut, Storrs, CT, USA

[2] Silvestrum Climate Associates, Woods Hole, MA, USA

[3] U.S. Geological Survey, Reston, VA, USA

[4] U.S. Fish and Wildlife Service, Vancouver, WA, USA

[5] College of Science and Engineering, James Cook University, Townsville, Australia

* xiucheng.yang@uconn.edu; zhe@uconn.edu

Contents of Tables

Table S-1. Definition of ac/deceleration 1
Table S-2. Statistics of the interpreted drivers 2
Table S-3. Interpretation of the drivers 3
Table S-4. Estimates of accuracy, area, and variance for the tidal wetland cover maps 4
Table S-5. Estimates of accuracy, area, and variance for the tidal wetland cover change. 5

Contents of Figures

Figure S-1. Conceptual comparison of epoch-based and dense time-series approaches 6
Figure S-2. Annual distribution of tidal marsh in different US coasts 7
Figure S-3. Illustration of sea level rise and constant value of DEM elevation 8
Figure S-4. Examples of tidal wetland permanent loss. 9
Figure S-5. Examples of tidal wetland permanent gain. 10
Figure S-6. Examples of tidal wetland fluctuation. 11
Figure S-7. Compounded impact from continuous hurricanes. 12
Figure S-8. Uncertainty of the map prediction on permanent loss 13
Figure S-9. Estimate of annual gain or loss trend and strength of trend. 14
Figure S-10. Trend of tidal marsh declining from 1985 to 2023 15

Table S-1. Definition of ac/deceleration based on annual gain/loss trend and the strength of trend.

		Strength of trend ($km^2 \cdot year^{-2}$)	
		Positive value	Negative value
Annual gain/loss trend ($km^2 \cdot year^{-1}$)	Positive value	**Accelerated gain**	**Decelerated gain**
	Negative value	**Decelerated loss**	**Accelerated loss**

Table S-2. Statistics of the interpreted drivers with bootstrapping uncertainty analysis for US tidal wetland cover changes. Uncertainty is estimated by using 1000 times of bootstrapping with a 95% confidence interval.

	**Period**	**Number of the sample**			**Weights of each sample**	**Contribution to cumulative cover change**		
		Extreme weather events	Direct human activities	Chronic stressors		Extreme weather events	Direct human activities	Chronic stressors
Permanent loss	1986-1990	5.0 [2.0 8.0]	1.9 [0.0 4.0]	6.1 [3.0 10.0]	14879	5.8 [4.7 6.2]	2.3 [0.0 3.2]	7.1 [7.1 7.2]
	1991-1995	1.0 [0.0 3.0]	0.0 [0.0 0.0]	9.2 [6.0 13.0]	10850	6.6 [4.7 7.8]	2.3 [0.0 3.2]	15.0 [14.1 17.3]
	1996-2000	0.0 [0.0 0.0]	2.0 [0.0 4.0]	6.0 [3.0 9.0]	12121	6.6 [4.7 7.8]	4.2 [0.0 5.8]	20.7 [19.7 23.1]
	2001-2005	5.1 [2.0 8.0]	0.0 [0.0 0.0]	8.9 [5.0 13.0]	20568	14.8 [11.7 16.4]	4.2 [0.0 5.8]	35.0 [32.5 40.8]
	2006-2010	9.1 [6.0 13.0]	0.0 [0.0 0.0]	2.0 [0.0 4.0]	17960	27.6 [27.7 28.1]	4.2 [0.0 5.8]	37.8 [36.4 40.8]
	2011-2015	3.0 [1.0 6.0]	0.0 [0.0 0.0]	10.0 [6.0 14.0]	11582	30.3 [29.8 31.1]	4.2 [0.0 5.8]	46.9 [44.3 52.8]
	2016-2020	4.0 [1.0 7.0]	0.0 [0.0 0.0]	7.0 [4.0 11.0]	21554	37.1 [33.6 38.7]	4.2 [0.0 5.8]	58.7 [55.5 66.4]
Permanent gain	1986-1990	0.0 [0.0 0.0]	1.1 [0.0 3.0]	6.0 [3.0 9.5]	8673	0.0 [0.0 0.0]	1.7 [0.0 3.0]	9.4 [9.0 9.6]
	1991-1995	0.0 [0.0 0.0]	4.1 [1.0 7.0]	7.9 [5.0 11.0]	6539	0.0 [0.0 0.0]	6.5 [2.5 8.4]	18.7 [17.5 21.0]
	1996-2000	0.0 [0.0 0.0]	8.0 [4.0 12.0]	6.1 [3.0 9.0]	6262	0.0 [0.0 0.0]	15.5 [13.0 16.8]	25.6 [24.5 27.8]
	2001-2005	1.0 [0.0 3.0]	1.0 [0.0 3.0]	9.0 [5.0 13.0]	5234	1.0 [0.0 1.8]	16.4 [13.0 18.5]	34.1 [32.0 38.5]
	2006-2010	1.0 [0.0 3.0]	9.9 [6.0 14.0]	4.1 [1.0 7.0]	7119	2.2 [0.0 4.2]	29.3 [29.3 29.6]	39.4 [37.9 41.2]
	2011-2015	0.0 [0.0 0.0]	7.0 [4.0 11.0]	6.0 [3.0 9.0]	6604	2.2 [0.0 4.2]	37.6 [37.6 38.5]	46.5 [44.8 48.3]
	2016-2020	0.0 [0.0 0.0]	3.9 [1.0 7.0]	8.1 [5.0 12.0]	6294	2.2 [0.0 4.2]	42.1 [40.9 42.6]	55.7 [53.2 59.1]
Fluctuation	1986-1990	13.0 [9.0 16.0]	0.0 [0.0 0.0]	3.0 [1.0 6.0]	11883	18.7 [16.2 23.0]	0.0 [0.0 0.0]	4.3 [2.2 5.8]
	1991-1995	9.0 [6.0 13.0]	1.0 [0.0 3.0]	5.0 [2.0 8.0]	6211	25.5 [22.8 30.2]	0.7 [0.0 1.5]	8.1 [4.7 10.2]
	1996-2000	13.0 [9.0 17.0]	0.0 [0.0 0.0]	3.0 [1.0 6.0]	8491	38.9 [34.5 46.5]	0.7 [0.0 1.5]	11.2 [6.3 14.3]
	2001-2005	11.1 [7.0 15.0]	0.0 [0.0 0.0]	4.0 [1.0 7.0]	7255	48.6 [43.4 57.4]	0.7 [0.0 1.5]	14.7 [7.8 18.7]
	2006-2010	12.0 [8.0 15.0]	0.0 [0.0 0.0]	3.0 [1.0 6.0]	8135	60.4 [53.8 71.1]	0.7 [0.0 1.5]	17.7 [9.3 22.7]
	2011-2015	6.0 [3.0 10.0]	0.0 [0.0 0.0]	2.9 [1.0 6.0]	3448	62.9 [56.6 73.1]	0.7 [0.0 1.5]	18.9 [9.9 24.4]
	2016-2020	11.0 [7.0 15.0]	0.0 [0.0 0.0]	6.0 [3.0 10.0]	8485	74.2 [67.0 85.1]	0.7 [0.0 1.5]	25.1 [14.9 31.5]

Table S-3. Interpretation of the drivers for tidal wetland cover changes. Uncertainty estimates are conducted by using 1000 times of bootstrapping under 95% confidence interval.

Driver	Tidal wetland			Tidal marsh			Mangrove			Tidal flat		
	Cumulative change proportion (%)											
	Permanent loss	Permanent gain	Fluctuation	Permanent loss	Permanent gain	Fluctuation	Permanent loss	Permanent gain	Fluctuation	Permanent loss	Permanent gain	Fluctuation
Extreme weather events	37.1 [33.6 38.7]	2.2 [0.0 4.2]	74.2 [67.0 85.1]	29.1 [22.1 32.2]	2.8 [0.0 5.0]	82.3 [77.7 100.0]	100.0 [100.0 100.0]	-	98.8 [97.5 100.0]	-	-	-
Direct human activities	4.2 [0.0 5.8]	42.1 [40.9 42.6]	0.7 [0.0 1.5]	2.9 [0.0 4.6]	50.2 [48.3 55.7]	-	-	-	1.2 [0.0 2.5]	-	-	-
Chronic stressors	58.7 [55.5 66.4]	55.7 [53.2 59.1]	25.1 [14.9 31.5]	68.0 [63.2 77.9]	47.0 [44.3 46.8]	17.7 [0.0 22.3]	-	100.0 [100.0 100.0]	-	-	-	-
Total	100.00	100.00	100.00	100.00	100.00	100.00	100.00	100.00	100.00	-	-	-
	Strength of trend on cumulative change proportion (% yr^{-2})											
	Permanent loss	Permanent gain	Fluctuation	Permanent loss	Permanent gain	Fluctuation	Permanent loss	Permanent gain	Fluctuation	Permanent loss	Permanent gain	Fluctuation
Extreme weather events	0.23 [0.15 0.24]	-	0.18 [0.15 0.19]	0.10 [0.00 0.13]	-	-0.21 [-0.28 0.00]	1.84 [0.00 1.89]	-	0.33 [0.28 0.39]	-	-	-
Direct human activities	-	-0.01 [-0.02 -0.01]	-0.03 [-0.06 0.00]	-	-0.02 [-0.02 -0.01]	-	-	-	-0.05 [-0.10 0.00]	-	-	-
Chronic stressors	0.16 [0.14 0.17]	-0.01 [-0.02 -0.00]	0.10 [0.10 0.11]	0.26 [0.23 0.30]	-0.12 [-0.16 -0.09]	-	-	0.75 [0.54 1.49]	-	-	-	-
Total	0.39	-0.02	0.25	0.36	-0.14	-0.21	1.84	0.75	0.28	-	-	-

Table S-4. Confusion matrices and estimates of accuracy, area, and variance for the tidal wetland cover maps. The error matrices (95% CI) are from the stratified random sample for the entire study period (1985-2023) expressed as area proportions and sample counts in addition to accuracy and area estimates from the stratified estimator. Columns represent reference labels and area estimates and rows represent map strata.

	Reference data					
Map class	Tidal marsh	Mangrove	Dieback	Tidal flats	Open water	Others
**Confusion matrix, sample counts**						
Tidal marsh	140	0	0	0	1	7
Mangrove	0	150	1	0	0	0
Dieback	0	0	153	0	0	0
Tidal flats	1	0	0	143	0	8
Open water	0	1	0	0	824	0
Others	2	0	0	0	0	597
**Confusion matrix, area proportions**						
Tidal marsh	0.0543	0.0000	0.0000	0.0000	0.0004	0.0027
Mangrove	0.0000	0.0074	0.0000	0.0000	0.0000	0.0000
Dieback	0.0000	0.0000	0.0001	0.0000	0.0000	0.0000
Tidal flats	0.0000	0.0000	0.0000	0.0046	0.0000	0.0003
Open water	0.0000	0.0007	0.0000	0.0000	0.5369	0.0000
Others	0.0013	0.0000	0.0000	0.0000	0.0000	0.3912
**Accuracy and area estimates**						
Map bias [%]	+3.2 [-1.0 +7.7]	-7.5 [-18.4 +6.8]	-28.7 [-51.5 +35]	+6.3 [+2.8 +10]	0 [-0.2 +0.3]	-0.4 [-1 +0.2]
User’s acc. [%]	94.59 ± 3.07	99.34 ± 1.09	100.00 ± 0.00	94.08 ± 3.16	99.88 ± 0.20	99.67 ± 0.39
Producer’s acc. [%]	97.59 ± 2.67	91.91 ± 12.23	71.34 ± 33.63	100.00 ± 0.00	99.93 ± 0.12	99.25 ± 0.42
Overall acc. [%]	99.46 ± 0.26					

Table S-5. Confusion matrices and estimates of accuracy, area, and variance for the tidal wetland cover change. The error matrices (95% CI) are derived from a stratified random sample aggregated over the entire study period (1986–2021) and are expressed as sample counts and area-weighted proportions. Accuracy and area estimates are obtained using a design-based stratified estimator, with class-specific area correction rates used to adjust mapped change areas. Columns represent reference change labels and area estimates, and rows represent map-derived change strata.

		**Reference data**		
Map class	Loss	Gain	Fluctuations	Others
		**Confusion matrix, sample counts**		
Loss	81	0	10	49
Gain	0	83	3	54
Fluctuations	2	2	103	33
Others	0	0	0	420
		**Confusion matrix, area proportions**		
Loss	0.0085	0	0.0011	0.0048
Gain	0	0.0036	0.0001	0.0024
Fluctuations	0.0001	0.0001	0.0054	0.0016
Others	0	0	0	0.9723
		**Accuracy and area estimates**		
User's acc. [%]	59.06 ± 8.47	58.54 ± 8.29	75.76 ± 7.45	100.00 ± 0.00
Producer's acc. [%]	98.97 ± 1.23	98.26 ± 2.10	81.58 ± 8.63	99.10 ± 0.14
Overall acc. [%]	98.98 ± 0.14			

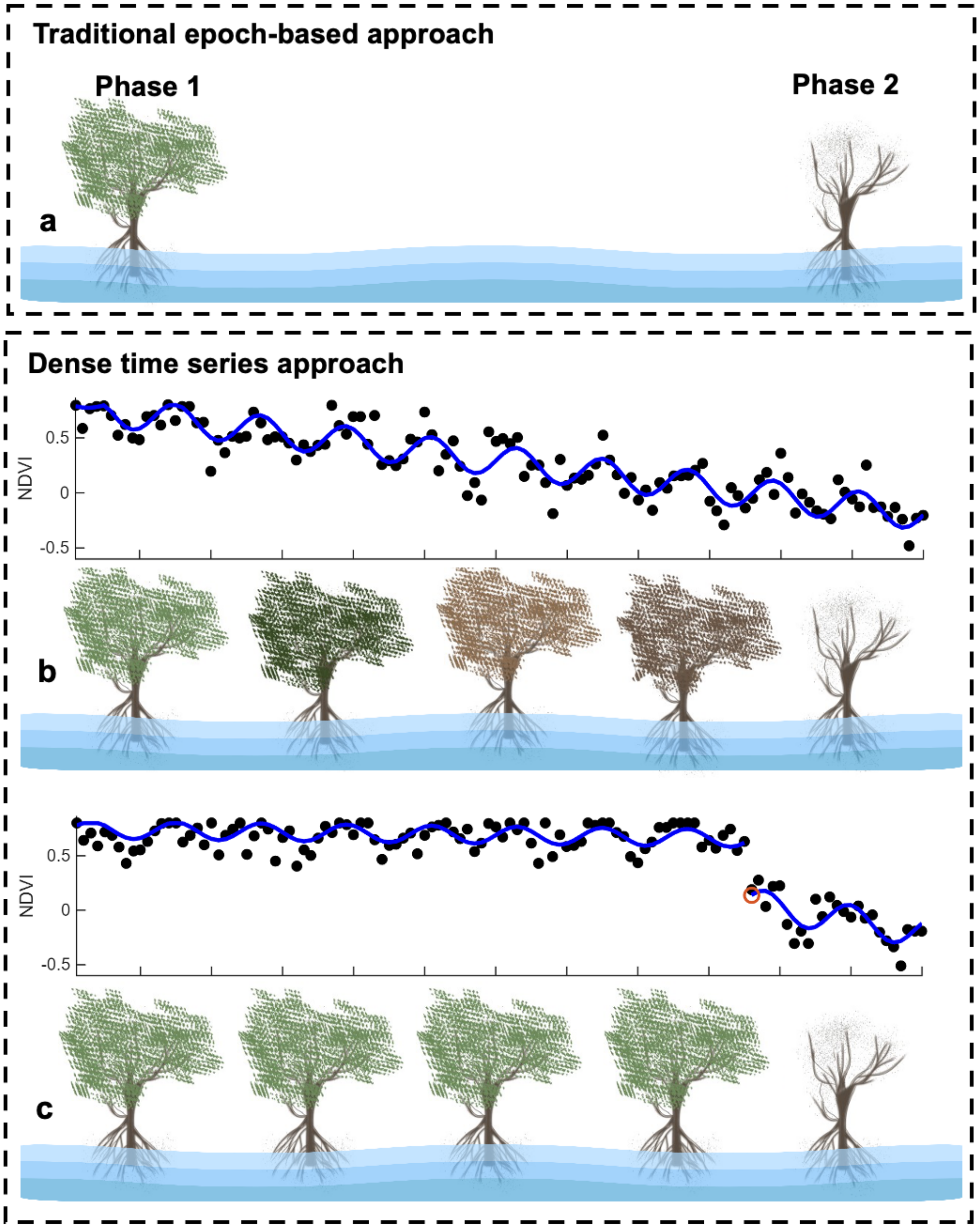

Figure S-1. Conceptual comparison of epoch-based and dense time-series approaches for detecting tidal wetland change. a, Epoch-based mapping compares land cover at two disconnected time slices and identifies net change, but provides limited information on the timing or underlying processes driving that change**. b, c,** Dense time-series approach tracks wetland condition continuously through time, enabling differentiation between gradual change driven by chronic stressors (**b**) and abrupt change triggered by acute pulse events (**c**). See Figure S4–S6 for real Landsat time-series examples of gains and losses associated with chronic and acute drivers.

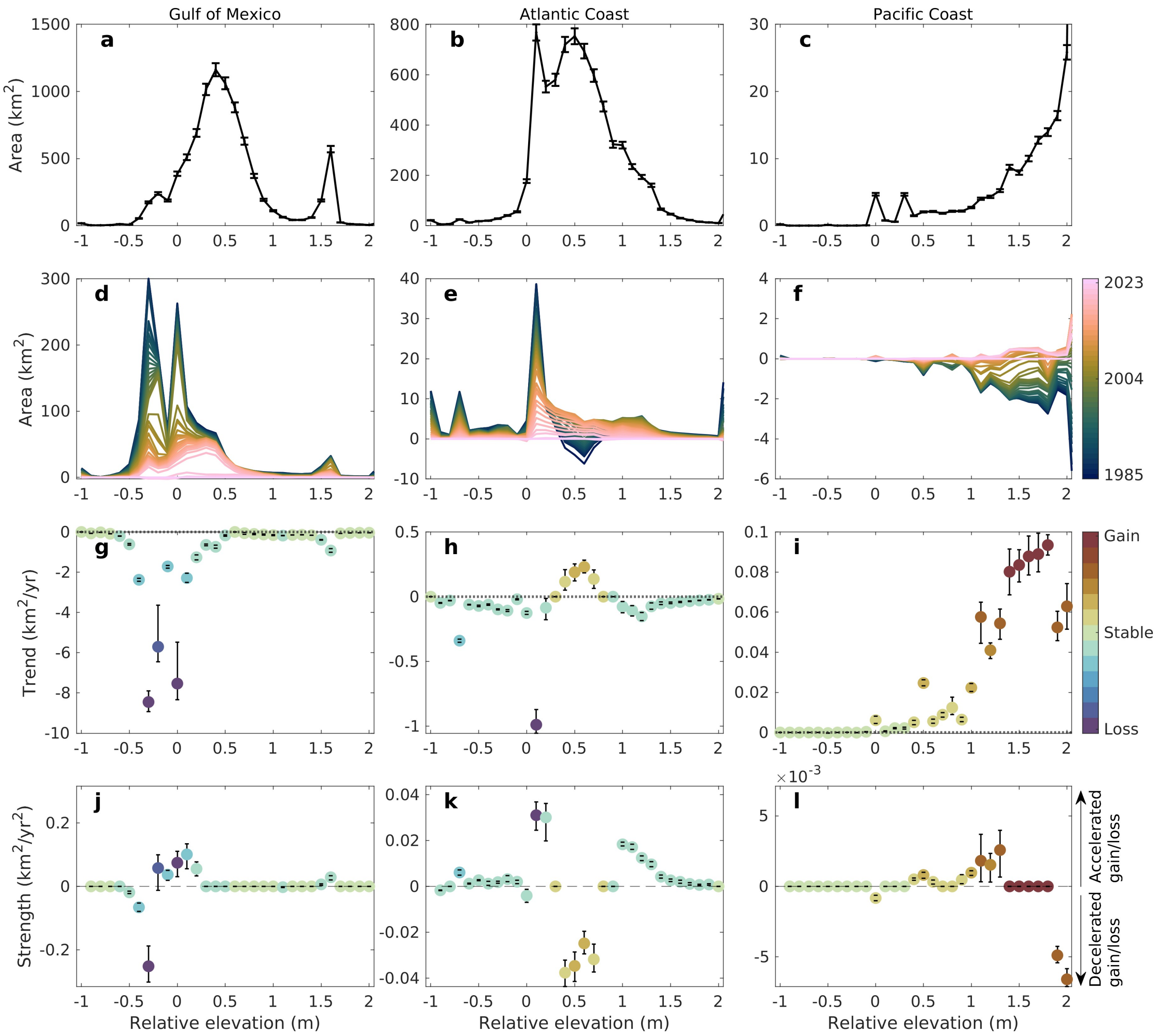

Figure S-2. Annual distribution of tidal marsh in different US coasts (Gulf of Mexico, Atlantic Coast and Pacific Coast), parsed by elevation. a-c, Area of tidal marsh in 2023 in 0.1 m elevation bins. **d-f,** the difference in the yearly extent of tidal marshes relative to the 2023 area within each 0.1 elevation interval. **g-i,** Annual gain or loss trend of the tidal marsh for every 0.1 m elevation range in different coastal regions. **j-l,** Acceleration, and deceleration of the gain and loss trends for every 0.1 m elevation range in different coastal regions (Refer to Table S-1). Positive trend strength indicates either decreasing losses or increasing gains of tidal marsh annually. Negative trend strength indicates either increasing losses or decreasing gain of tidal marsh annually. The elevation data are sourced from the USGS 3D Elevation Program (3DEP) Dataset, collected from around 2000 to 2015, and is referenced to the North American Vertical Datum of 1988 (NAVD88). That is, the elevation is relative values based on a static state, and not changed with sea level rise in the study period (Refer to Figure S-3). Y-axis scales vary by region.

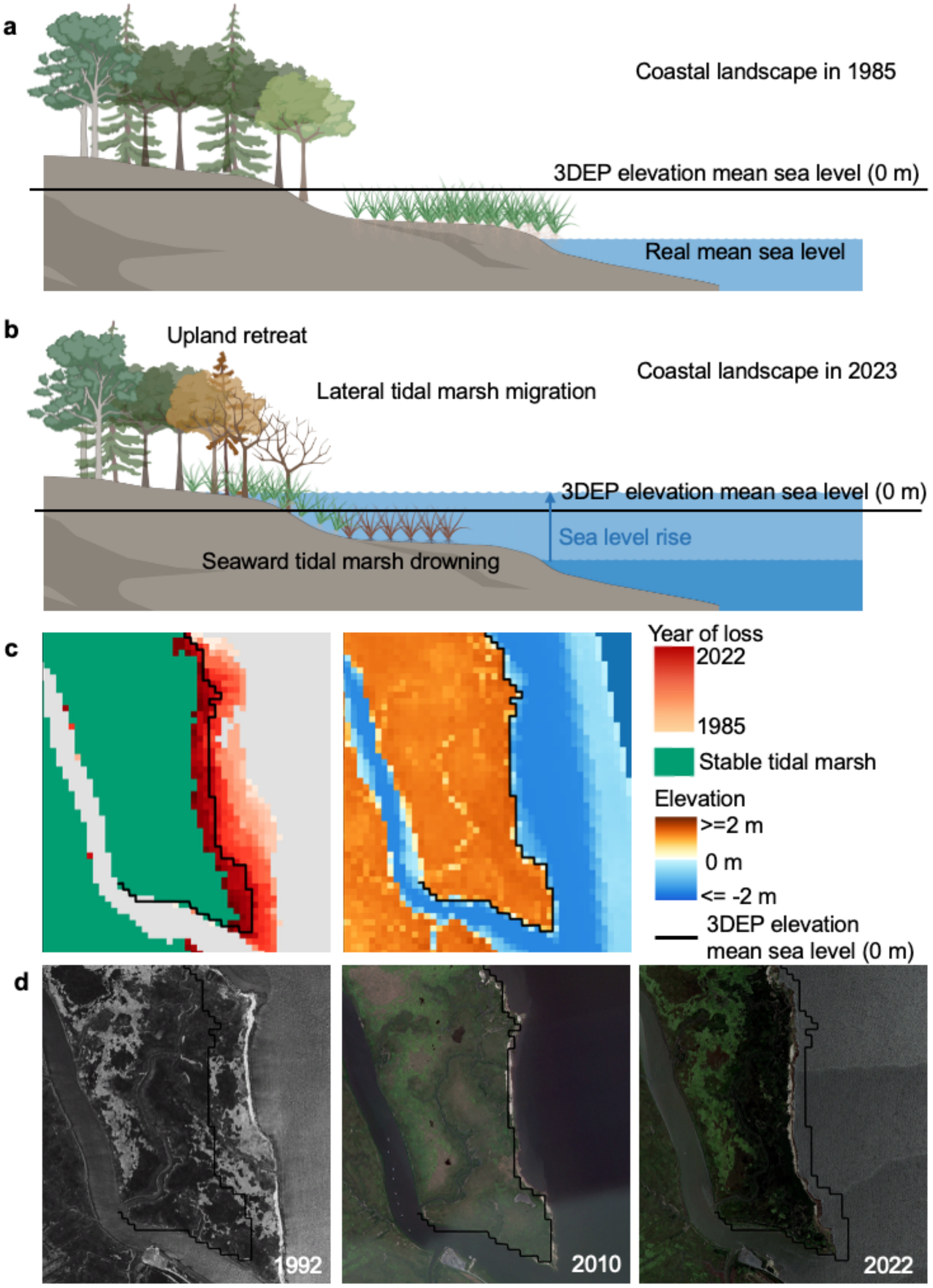

Figure S-3. Illustration of sea level rise and constant value of DEM elevation. **a, b,** Illustration of the difference between the real mean sea level and elevation data derived from USGS 3D Elevation Program (3DEP). Created in BioRender. Yang, X. (2026) https://BioRender.com/a0vnmzp. **c,** 3DEP elevation data is static with the conditions during its generation. Thus, for the regions that existed tidal marsh before would have a negative elevation range with sea level rise. **d,** Historic very high resolution from Google Earth to show the process of sea level rise. Location: 39.2° N, -75.4° W.

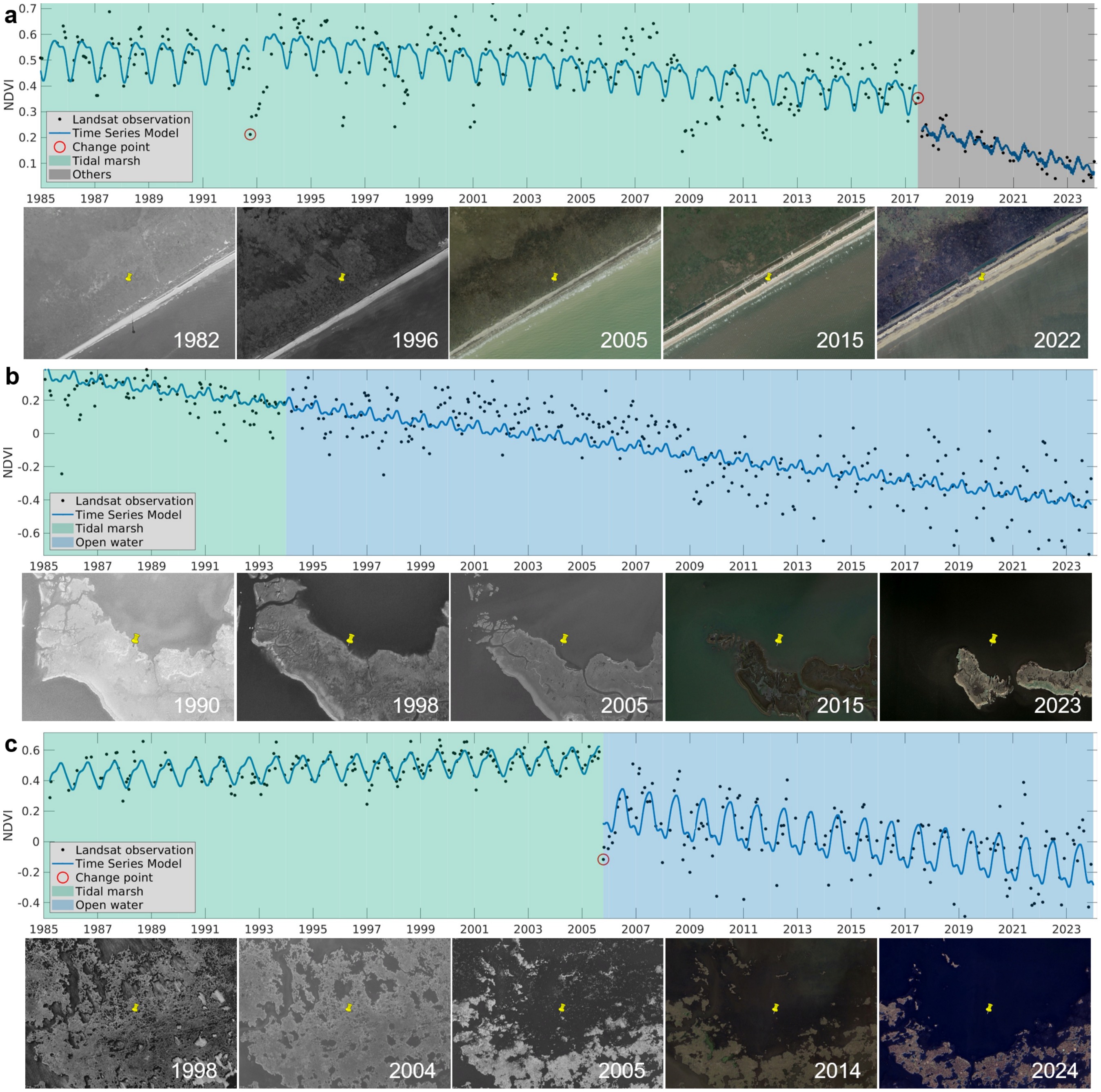

Figure S-4. Examples of tidal wetland permanent loss. a, Tidal marsh loss due to chronic stressors of coastal erosion (29.64° N, 94.16° W). **b,** Tidal marsh drowning dominated by chronic stressors of relative sea level rise (29.31° N, 90.52° W). **c,** Hurricane Katrina that led to the immediate loss of previously stable tidal marsh (29.76° N, 89.77° W).

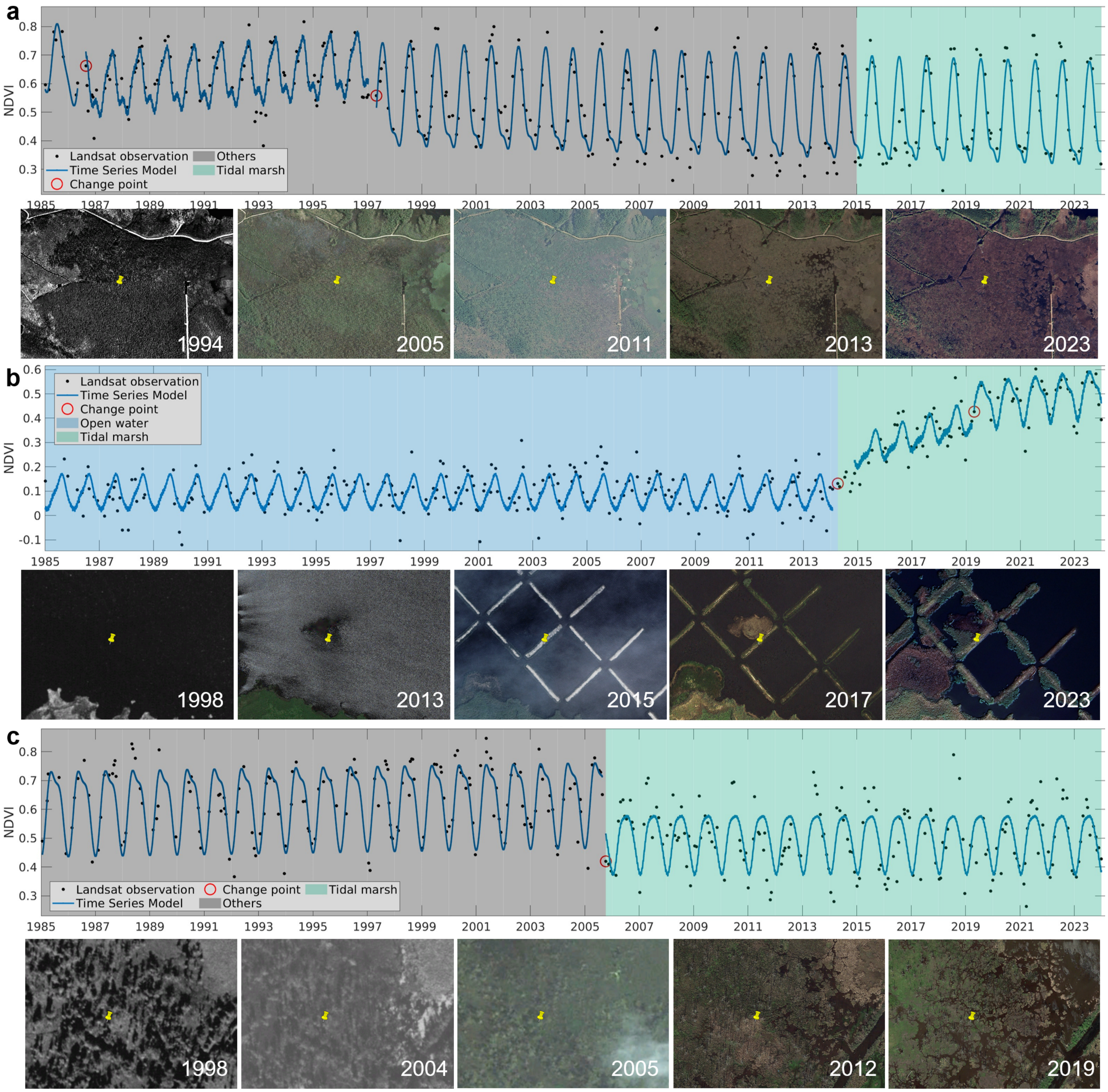

Figure S-5. Examples of tidal wetland permanent gain. a, Tidal marsh landward migration due to chronic stressor of sea level rise (38.44° N, 76.21° W). **b,** Direct human activities of tidal marsh restoration (29.97° N, 93.51° W). **c,** Hurricane Katrina benefit the tidal marsh migration due to the removal of upland woody forests and irregular tidal intrusion (29.51° N, 90.64° W).

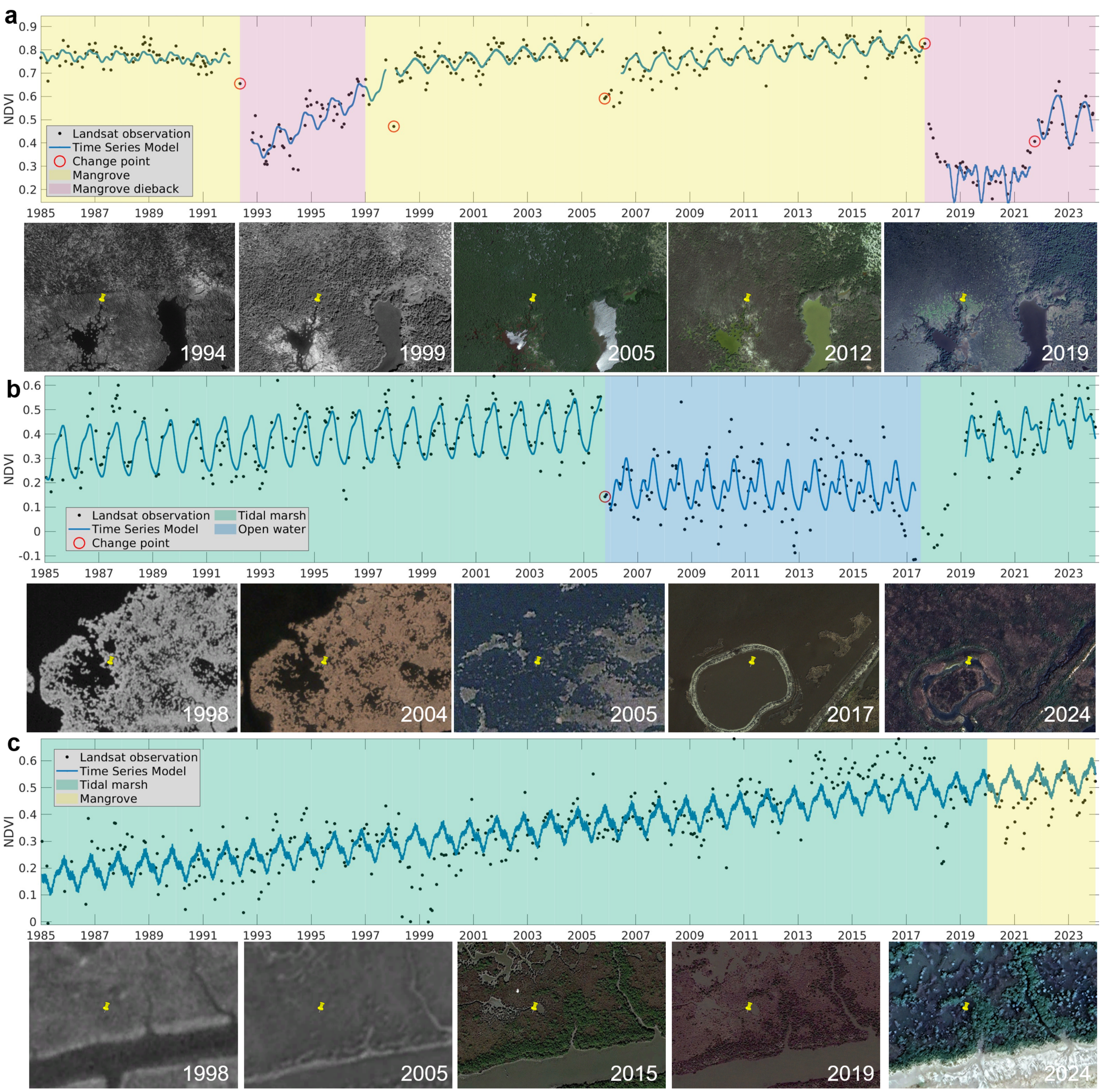

Figure S-6. Examples of tidal wetland fluctuation. a, Temporal mangrove loss with dieback and recovery due to Hurricanes Andrew in 1992, Wilma in 2005 and Irma in 2017 (25.63° N, 81.23° W). **b,** Temporal tidal marsh loss due to hurricane Katrina and recovery with restoration (30.24° N, 89.85° W). **c,** Internal transitions with mangrove encroachment to tidal marsh (29.05° N, 90.73° W).

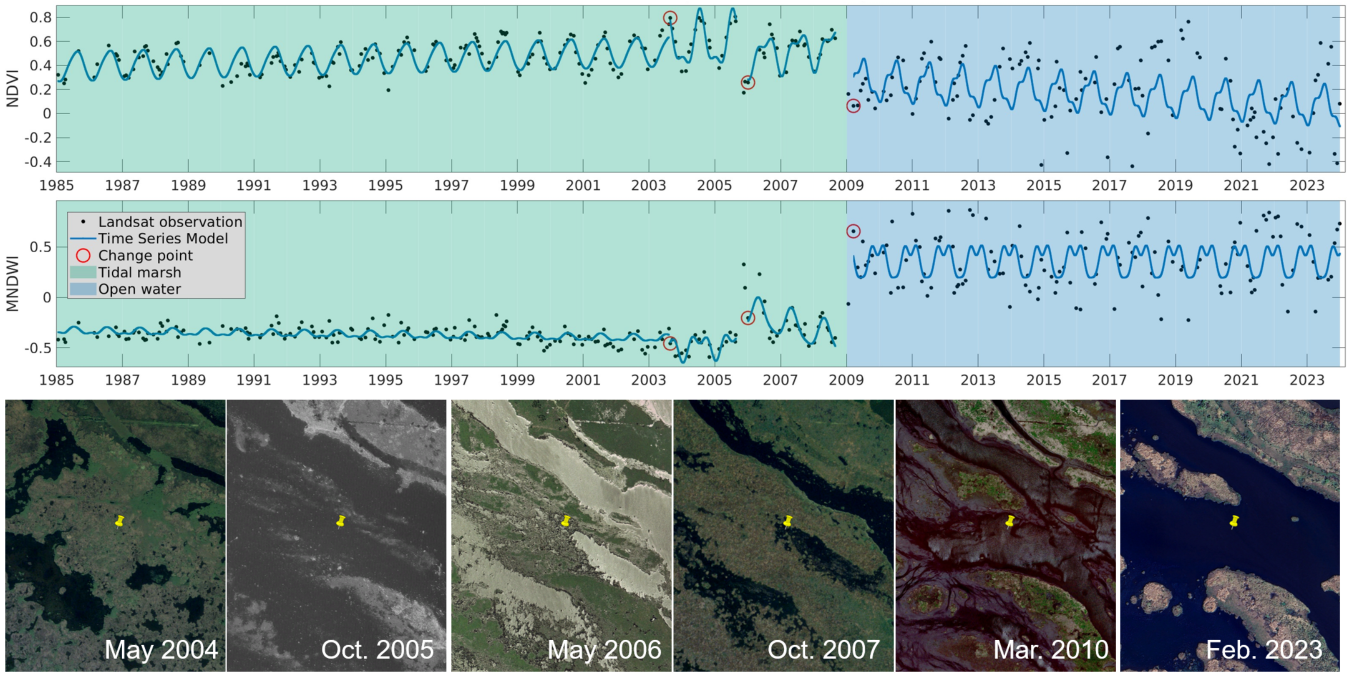

Figure S-7. Compounded impact from continuous hurricanes. The tidal marsh pixel located in the Mississippi River Delta (29.78° N, 89.96° W) experienced significant ecological disturbances due to Hurricane Katrina in 2005 and Hurricane Gustav in 2008. The impact of Hurricane Katrina led to a noticeable degradation in the tidal marsh condition. By 2008, the tidal marsh had not yet fully recovered to its pre-disturbance state. The subsequent hit from Hurricane Gustav pushed the marsh beyond its tipping point, resulting in a permanent loss of this critical habitat.

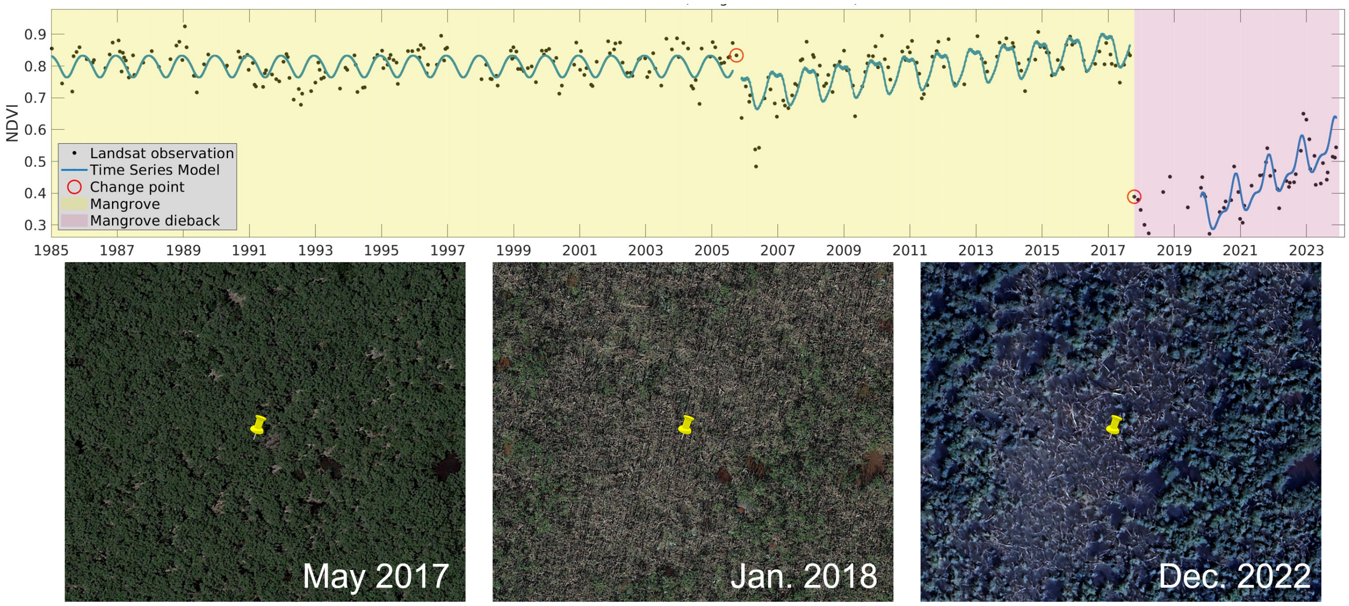

Figure S-8. Uncertainty of the map prediction on permanent loss due to the undergoing recovery period at the end of the study period. In the sample-based analysis, although there is a permanent loss of mangrove predicted in the map in 2017 due to Hurricane Irma (25.93° N, 81.55° W), we interpret the change type as fluctuation to correct the lag due to the recovery process. In this way, we can exclude the "false permanent loss" in our driver analysis.

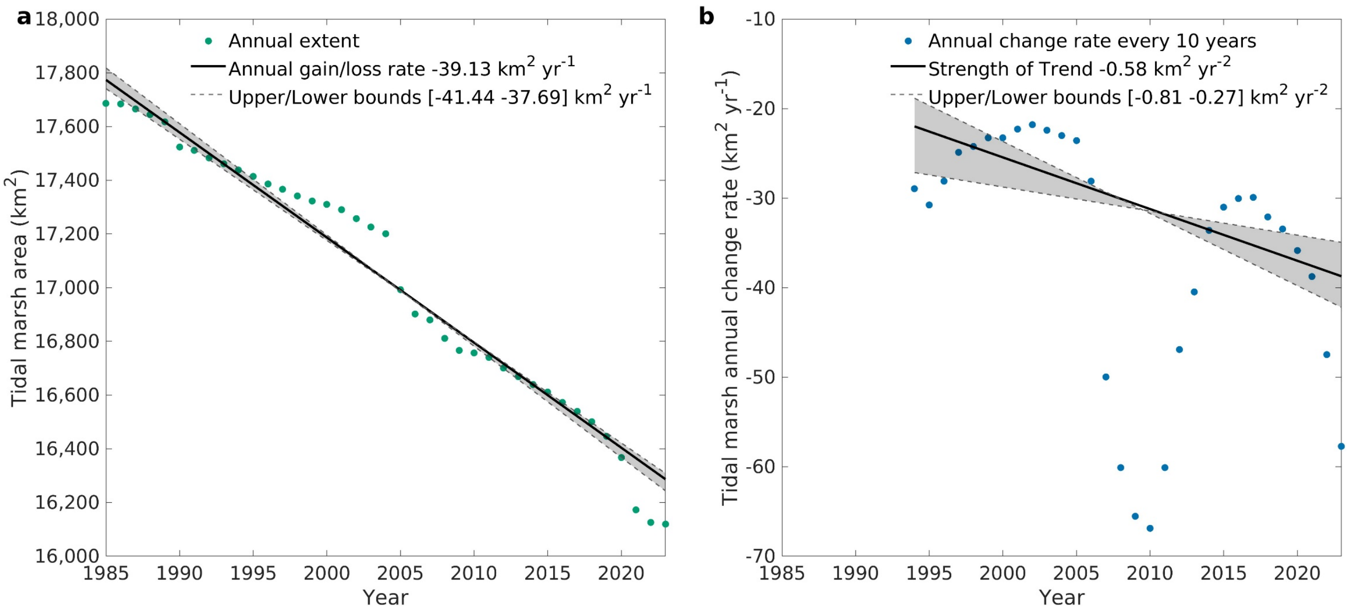

Figure S-9. Estimate of annual gain or loss trend and strength of trend. The example is based on the annual area of tidal marsh in the conterminous US. **a**, Annual gain or loss trend based on the Sen's slope and MK-test (95% confidence intervals). **b**, Strength of trend estimated with a 10-year moving window. Each blue point indicates the annual change rate calculated within a 10-year period, beginning at the period of 1985-1994. Then, 30 annual change rates are calculated for each temporal window, and a subsequent Sen's slope and MK-test (95% confidence intervals) analysis is used to calculate the strength of the trend (i.e., acceleration or deceleration of the annual change rate). Here, both the negative values of the annual trend and strength of trend indicate that the accelerated loss of the tidal marsh in the conterminous US (refer to Table S-1).

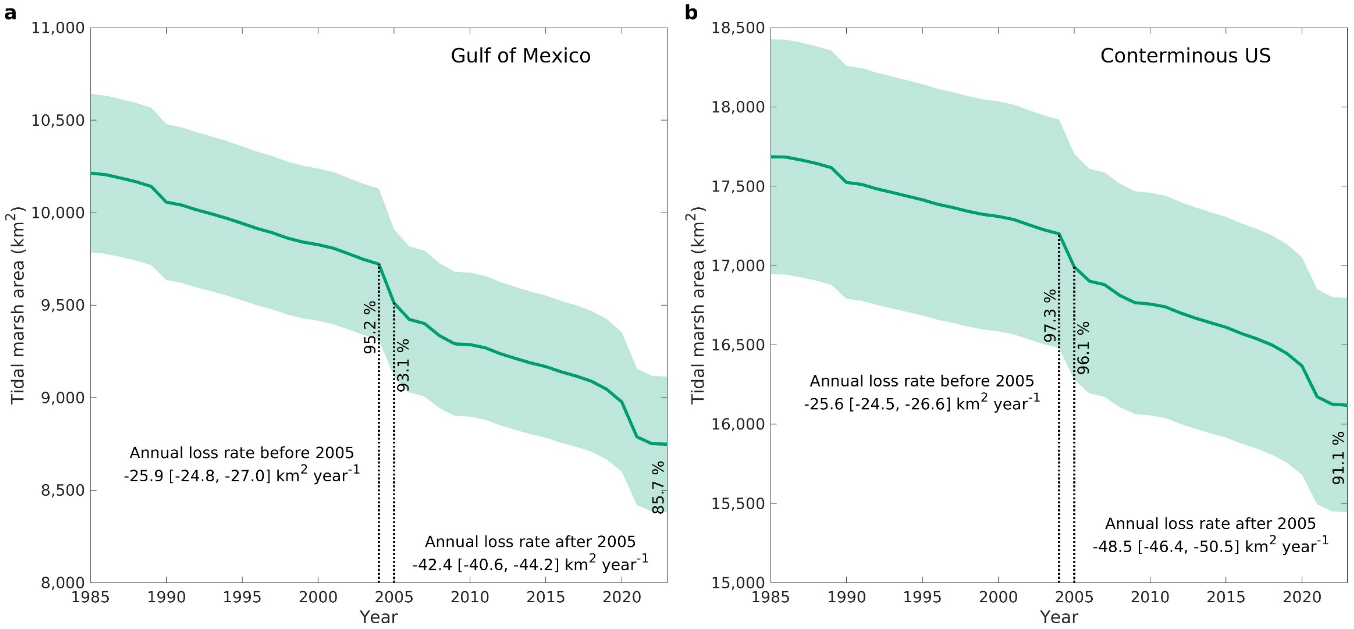

Figure S-10. Trend of tidal marsh declining from 1985 to 2023. a, b, Trends of tidal marsh in Gulf of Mexico and conterminous US, respectively. Since 2005, the occurrence of compounded events including Hurricanes Katrina and Rita in 2005, Hurricane Gustav in 2008, and Hurricane Laura and Ida in 2020 and 2021, along with the continuously increasingly powerful chronic stressors of accelerated sea level rise, the loss speed of the tidal marsh in the US is almost doubled from 2005. Values on the vertical lines indicate percentage and uncertainty of 1986 coverage remaining.
