# Supplementary material for: The accelerating loss and shifting dynamics of US tidal wetlands
Source: Nat Commun. 2026 May 19;17:4332. doi: 10.1038/s41467-026-71464-2 (PMC13187131; doi:10.1038/s41467-026-71464-2)
Supplement: Supplementary file 2 — Reporting Summary [file 41467_2026_71464_MOESM2_ESM.pdf]

nature portfolio

Corresponding author(s): Xiucheng Yang, Zhe Zhu

Last updated by author(s): Jan 30, 2026

Reporting Summary

Nature Portfolio wishes to improve the reproducibility of the work that we publish. This form provides structure for consistency and transparency in reporting. For further information on Nature Portfolio policies, see our Editorial Policies and the Editorial Policy Checklist.

Statistics

For all statistical analyses, confirm that the following items are present in the figure legend, table legend, main text, or Methods section.

n/a	Confirmed	
☐	☒	The exact sample size (*n*) for each experimental group/condition, given as a discrete number and unit of measurement
☐	☒	A statement on whether measurements were taken from distinct samples or whether the same sample was measured repeatedly
☐	☒	The statistical test(s) used AND whether they are one- or two-sided<br>*Only common tests should be described solely by name; describe more complex techniques in the Methods section.*
☐	☒	A description of all covariates tested
☐	☒	A description of any assumptions or corrections, such as tests of normality and adjustment for multiple comparisons
☐	☒	A full description of the statistical parameters including central tendency (e.g. means) or other basic estimates (e.g. regression coefficient) AND variation (e.g. standard deviation) or associated estimates of uncertainty (e.g. confidence intervals)
☐	☒	For null hypothesis testing, the test statistic (e.g. *F*, *t*, *r*) with confidence intervals, effect sizes, degrees of freedom and *P* value noted<br>*Give P values as exact values whenever suitable.*
☒	☐	For Bayesian analysis, information on the choice of priors and Markov chain Monte Carlo settings
☒	☐	For hierarchical and complex designs, identification of the appropriate level for tests and full reporting of outcomes
☒	☐	Estimates of effect sizes (e.g. Cohen's *d*, Pearson's *r*), indicating how they were calculated

Our web collection on statistics for biologists contains articles on many of the points above.

Software and code

Policy information about availability of computer code

Data collection	We used Python API to batch downloading the historic Landsat satellite data from USGS.
Data analysis	We used Matlab to conduct the experiments and analyses. All codes have been released in GitHub.

For manuscripts utilizing custom algorithms or software that are central to the research but not yet described in published literature, software must be made available to editors and reviewers. We strongly encourage code deposition in a community repository (e.g. GitHub). See the Nature Portfolio guidelines for submitting code & software for further information.

Data

Policy information about availability of data

All manuscripts must include a data availability statement. This statement should provide the following information, where applicable:

- Accession codes, unique identifiers, or web links for publicly available datasets
- A description of any restrictions on data availability
- For clinical datasets or third party data, please ensure that the statement adheres to our policy

All data used in this study were obtained from open data sources. The Landsat Collection 2 US Analysis Ready Data (ARD) data were downloaded from USGS https://earthexplorer.usgs.gov/. Tide predictions were obtained from global ocean tide models (EOT20) developed at DGFI-TUM https://www.seanoe.org/data/00683/79489/. Historical maps of the NOAA C-CAP Regional Land Cover and Change maps were obtained from https://coast.noaa.gov/digitalcoast/data/ccapregional.html. The seamless 3DEP DEM dataset for the US were provided by USGS (https://www.usgs.gov/3d-elevation-program) and downloaded from Google Earth Engine (https://developers.google.com/earth-engine/datasets/catalog/USGS_3DEP_10m). National Wetlands Inventory (NWI) dataset (https://www.fws.gov/

program/national-wetlands-inventory) is employed to help interpret the training and validation data. To interpret the drivers associated with the extreme weather events, historical hurricane tracks (https://coast.noaa.gov/hurricanes/) and National Integrated Drought Information System (NIDIS) (https://www.drought.gov/) in NOAA platforms are used. Tidal wetland annual extent, loss and gain, and change derived from our DECODE approach and supporting our analyses are available at https://gers.users.earthengine.app/view/tidalwetlandcover.

Research involving human participants, their data, or biological material

Policy information about studies with human participants or human data. See also policy information about sex, gender (identity/presentation), and sexual orientation and race, ethnicity and racism.

Reporting on sex and gender	none
Reporting on race, ethnicity, or other socially relevant groupings	none
Population characteristics	none
Recruitment	none
Ethics oversight	none

Note that full information on the approval of the study protocol must also be provided in the manuscript.

Field-specific reporting

Please select the one below that is the best fit for your research. If you are not sure, read the appropriate sections before making your selection.

☐ Life sciences ☐ Behavioural & social sciences ☒ Ecological, evolutionary & environmental sciences

For a reference copy of the document with all sections, see nature.com/documents/nr-reporting-summary-flat.pdf

Ecological, evolutionary & environmental sciences study design

All studies must disclose on these points even when the disclosure is negative.

Study description	We analyzed annual tidal wetland dynamics across the conterminous United States (1985–2023) using the full Landsat archive. A dense time-series change detection framework (DECODE), random forest classification and sample-based analyses were used to quantify wetland extent, trends, acceleration, and drivers of change.
Research sample	The sample is from all mapped tidal wetlands (tidal marsh, mangrove, tidal flats, mangrove dieback) within US coastal landscapes, defined using NOAA C-CAP maps and USGS elevation data. Training and validation samples were derived from visually interpreted Landsat time series and high-resolution imagery.
Sampling strategy	Random sampling was applied for training, accuracy assessment, and driver attribution. Sample sizes were chosen to ensure representation across regions, wetland types, and change classes.
Data collection	Data were derived from Landsat 4–8 surface reflectance imagery (1985–2023) using automated batch downloading from USGS.
Timing and spatial scale	Data span 1985–2023 at annual temporal resolution and 30 m spatial resolution, covering the entire coastal landscape of the conterminous United States.
Data exclusions	No data were excluded based on results.
Reproducibility	All analyses used standardized, automated workflows available at GitHub (https://github.com/GERSL/DECODE). Change detection parameters were calibrated independently, and uncertainties were quantified using confusion matrices and bootstrapping.
Randomization	Simple randomization is used for training data collection. Stratified random sampling is used for uncertainty assessment and driver analyses.
Blinding	The reference sample was interpreted blind to map labels to avoid confirmation bias, on the reference of high-resolution historical images in Google Earth and time-series Landsat observations (1985–2024).

Did the study involve field work? ☐ Yes ☒ No

Reporting for specific materials, systems and methods

We require information from authors about some types of materials, experimental systems and methods used in many studies. Here, indicate whether each material, system or method listed is relevant to your study. If you are not sure if a list item applies to your research, read the appropriate section before selecting a response.

Materials & experimental systems

n/a	Involved in the study
☒	☐ Antibodies
☒	☐ Eukaryotic cell lines
☒	☐ Palaeontology and archaeology
☒	☐ Animals and other organisms
☒	☐ Clinical data
☒	☐ Dual use research of concern
☒	☐ Plants

Methods

n/a	Involved in the study
☒	☐ ChIP-seq
☒	☐ Flow cytometry
☒	☐ MRI-based neuroimaging

Plants

Seed stocks	none
Novel plant genotypes	none
Authentication	none
